# Familial Non-Medullary Thyroid Carcinoma: Distinct Clinicopathological Features and Prognostic Implications in a Large Cohort of 46,572 Patients

**DOI:** 10.3390/cancers17203381

**Published:** 2025-10-20

**Authors:** Cho Rok Lee, Jin Kyong Kim, Joon Ho, Sang-Wook Kang, Jandee Lee, Jong Ju Jeong, Kee-Hyun Nam, Woong Youn Chung

**Affiliations:** 1 Department of Surgery, Yongin Severance Hospital, Yonsei University College of Medicine, Yongin-si 16995, Republic of Korea; crlee@yuhs.ac (C.R.L.);; 2 Department of Surgery, Severance Hospital, Yonsei Cancer Center, Yonsei University College of Medicine, Seoul 03722, Republic of Koreajungjongj@yuhs.ac (J.J.J.);

**Keywords:** thyroid cancer, familial cancer, prognosis

## Abstract

**Simple Summary:**

Thyroid cancer has become increasingly common worldwide and can develop within families, in which case it is known as familial non-medullary thyroid cancer (FNMTC). We studied over 46,000 patients with thyroid cancer to understand how FNMTC differs from non-familial cases. We found that FNMTC occurs more often in women and at a younger age, and its prevalence has gradually risen over time. Although tumors in FNMTC were smaller, they showed higher rates of bilaterality, multifocality, and central lymph node metastasis. Family history was correlated with recurrence-free survival, especially in patients with other high-risk features. These findings suggest that consideration of family history may be warranted when formulating treatment strategies for patients in the intermediate-to-high-risk group.

**Abstract:**

**Background:** The incidence of thyroid cancer has rapidly increased worldwide, and familial aggregation of the disease has been increasingly recognized. This study aimed to evaluate the prevalence, clinicopathological characteristics, and long-term outcomes of familial non-medullary thyroid cancer (FNMTC) in a large institutional cohort. **Methods:** Patients with non-medullary thyroid cancer (NMTC) who had undergone surgery were classified as sporadic NMTC (SNMTC) or FNMTC based on family history. Clinicopathological features at diagnosis and surgery were compared, and prognostic outcomes were analyzed in patients with follow-up data. **Results:** Among the 46,572 NMTC patients, 3829 (8.2%) had FNMTC, and 42,743 (91.8%) had SNMTC. FNMTC was more prevalent in women and occurred at a younger age. Its proportion increased over time, peaking in the 35–59 age group. FNMTC showed higher rates of bilaterality (23.5% vs. 17.5%, *p* < 0.001), multifocality (39.0% vs. 30.5%, *p* < 0.001), and central lymph node metastasis (41.5% vs. 38.8%, *p* = 0.001), despite smaller tumors (0.9 ± 0.7 cm vs. 1.0 ± 0.9 cm, *p* < 0.001). Recurrence rates were similar between the two groups (1.9% vs. 2.3%, *p* = 0.1), but overall survival was higher in the FNMTC group (99.6% vs. 98.6%, *p* < 0.001). Family history, extracapsular extension, lymph node metastasis, and tumor size independently predicted recurrence. Family history significantly impacted recurrence-free survival in the intermediate-to-high-risk group (HR = 1.65, *p* < 0.001) but not in low-risk patients. **Conclusions:** FNMTC represents a distinct NMTC subset with more extensive local disease but favorable survival, warranting risk-adapted management, particularly for intermediate-to-high-risk patients.

## 1. Introduction

Over the past 20 years, the incidence of thyroid cancer has increased in different populations worldwide [1,2]. This trend is particularly pronounced in South Korea. From 1999 to 2021, the age-standardized incidence rates showed that thyroid cancer exhibited the greatest annual percentage change. Additionally, as of 2022, thyroid cancer had the highest prevalence—21.4%—among significant cancer types. This is attributable to its high incidence and exceptionally high survival rate [3,4]. Therefore, the appropriate management (surgery and follow-up) of thyroid cancer is essential.

The incidence of thyroid cancer subtypes varies across countries. However, in South Korea, differentiated thyroid carcinoma accounts for more than 95% of all cases. The cause of thyroid cancer remains unclear, but it is accepted that genetic and environmental factors affect the risk. Previous studies have reported that various risk factors for papillary thyroid carcinoma (PTC), such as radiation exposure, female sex, benign thyroid disease, high iodine intake, obesity, and a family history of PTC, are responsible for the increased prevalence of thyroid cancer [5,6,7,8].

A family history of PTC is reported as a risk factor for thyroid cancer. FNMTC is defined as differentiated thyroid cancer that occurs in at least two first-degree relatives, including the index patient, without other predisposing causes of thyroid cancer [9]. FNMTC, constituting 3–9% of all thyroid cancers [10,11], is further classified as nonsyndromic or syndromic, depending on whether the thyroid cancer component is the main cancer (nonsyndromic) in relatives or is one of many constellations of tumors (syndromic) in relatives. Syndromic FNMTC accounts for a minor proportion of familial cancer syndromes such as familial adenomatous polyposis, Gardner syndrome, and Cowden disease. Many susceptibility genes for syndromic FNMTC are known, but none have been identified for nonsyndromic FNMTC. Moreover, nonsyndromic FNMTC is much more common than syndromic FNMTC [12]. Due to the increasing incidence of thyroid cancer in the general population, some researchers have argued that the presence of NMTC in only two first-degree relatives could be a coincidental occurrence rather than evidence of a hereditary predisposition [13].

The natural progression of syndromic FNMTC resembles that of sporadic FNMTC. However, the aggressiveness of nonsyndromic FNMTC compared with that of sporadic NMTC remains a topic of debate. While some studies suggest that nonsyndromic FNMTC exhibits a more aggressive clinical course, others report no significant differences in disease severity, recurrence risk, or mortality compared with sporadic NMTC. Most studies have reported more aggressive disease at presentation with worse outcomes in the FNMTC group [14,15]. However, several studies have reported more aggressive disease at presentation with similar outcomes at the end of follow-up, or similar baseline characteristics with similar or worse outcomes [16,17,18,19,20]. Multiple studies have shown that patients with FNMTC tend to present with more advanced disease at diagnosis, which often leads to a more aggressive initial treatment [14,21,22,23,24]. However, there is no evidence to suggest that patients with FNMTC respond differently to surgery or radioactive iodine therapy (RAIT) than patients with sporadic thyroid cancer. Nevertheless, it is crucial to consider that while treatments for sporadic differentiated thyroid cancer (DTC) are trending toward less aggressive approaches, the applicability of these changes to families with FNMTC remains insufficiently evaluated.

In this study, we examined family histories of thyroid cancer in 46,572 patients who had visited our institution. We investigated the prevalence and clinical characteristics of FNMTC, including long-term outcomes, among a relatively large group of patients with NMTC.

## 2. Materials and Methods

### 2.1. Study Patients

This study retrospectively included patients who were pathologically confirmed to have differentiated thyroid carcinoma between January 1990 and December 2024 and were followed up at Severance Hospital in Yonsei University College of Medicine.

We directly asked the patients whether they had any first-degree relatives who had been diagnosed with thyroid cancer. Family history of thyroid cancer was assessed for all patients at the time of their first outpatient visit or hospital admission. The first-degree relatives included parents, offspring, and siblings. The FNMTC group included the patients whose first-degree relatives(s) had been diagnosed with thyroid cancer (differentiated thyroid cancer only) after operation or fine(core) needle aspiration biopsy. Patients whose first-degree relatives had been diagnosed with thyroid cancer other than differentiated thyroid cancer or whose relatives’ cancer subtype was unknown were excluded from this study. In families with multiple affected members, only the proband (the first diagnosed patient) was included in the analysis to avoid duplication. In this study, FNMTC was defined as the presence of at least one first-degree relative, other than the index patient, diagnosed with non-medullary thyroid carcinoma.

Patients with prior radiation exposure and coexisting anaplastic thyroid carcinoma, poorly differentiated thyroid carcinoma, medullary thyroid carcinoma, or other inherited familial cancer syndromes (e.g., familial adenomatous polyposis, Gardner’s syndrome, or Cowden’s disease) were excluded from this study. The presence of other inherited familial cancer syndromes was evaluated during the initial clinical assessment using a structured family history questionnaire. Patients who reported family histories suggestive of known hereditary cancer syndromes were subsequently excluded from the analysis to ensure a homogeneous study population. Among the 46,572 patients, 42,743 had sporadic NMTC, and 3829 had FNMTC.

To compare the clinicopathological characteristics of FNMTC and sporadic NMTC, the following parameters were examined and analyzed: age at the time of NMTC diagnosis, sex, histopathology, tumor size, lymph node metastasis, multiplicity, extrathyroidal extension, combined thyroid disease, treatment with radioactive iodine, staging, recurrence risk stratification, survival, and recurrence. Recurrence was defined as locoregional or distant, which was confirmed through histology or a whole-body scan and serum thyroglobulin following radioactive iodine therapy. Due to the complexity of analyzing a large heterogeneous cohort, patients were stratified according to ATA risk categories (low-risk group and intermediate-to-high-risk group) to facilitate more accurate and clinically relevant subgroup comparisons. We subdivided FNMTC into three groups for analysis according to the affected members (one, two, and three or more) and relationship (parent/offspring, sibling, and parent/offspring/siblings). For the analysis of long-term outcomes, patients who underwent surgery between 1990 and 2019 were selected and analyzed according to the ATA risk categories.

### 2.2. Statistical Analysis

All continuous variables were expressed as the means ± standard deviations (SDs). Statistical analyses were performed using Pearson’s chi-square test to compare categorical variables between the groups. One-way analysis of variance (ANOVA) was performed to assess the differences in means among the groups. Disease-free survival (DFS) was estimated using the Kaplan–Meier method, and survival curves were compared using the log-rank test. To adjust for potential confounding factors, we additionally performed univariate and multivariate Cox proportional hazards regression analyses to identify independent predictors of recurrence. Variables included in the multivariate model were age, sex, tumor size, surgical extent (lobectomy, subtotal, or total thyroidectomy), bilaterality, multiplicity, extracapsular extension, central and lateral lymph node metastasis, and family history. To further evaluate whether the effects of these risk factors varied by baseline recurrence risk, stratified analyses were performed according to the ATA risk categories (low-risk vs. intermediate-to-high-risk). Hazard ratios (HRs) and 95% confidence intervals (CIs) were calculated, and a *p*-value < 0.05 was considered statistically significant. All statistical analyses were performed using SAS version 9.4 (SAS Institute Inc., Cary, NC, USA) and Stata version 18.1 (Stata Corp LLC, College Station, TX, USA).

## 3. Results

### 3.1. Trends in Thyroid Cancer Surgery, Sex Ratio, and FNMTC Proportion (1990–2024)

**Figure 1:**
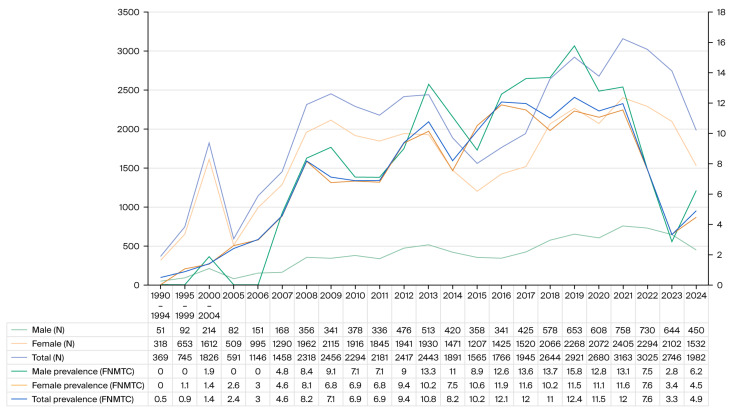
Trends in thyroid cancer surgery, sex ratio, and FNMTC proportion (1990–2024).

Figure 1 presents the number of patients who underwent surgery for thyroid cancer, the male-to-female ratio, and the proportion of FNMTC patients annually from 1990 to 2024. The number of patients with thyroid cancer increased sharply in the early 2000s and then showed a significant decline in 2014. However, approximately five years later, in 2018, the number of patients with thyroid cancer increased again. In 2024, the number of surgeries decreased due to a unique medical crisis in Korea’s medical policy. Additionally, the proportion of patients with FNMTC has gradually increased since the late 2000s. After 2014, when the total number of surgeries decreased, the proportion of FNMTC cases increased significantly, but it started to decline again in the 2020s. Regarding the sex ratio, the proportion of FNMTC cases has consistently increased in both sexes.

### 3.2. Prevalence of FNMTC

**Figure 2:**
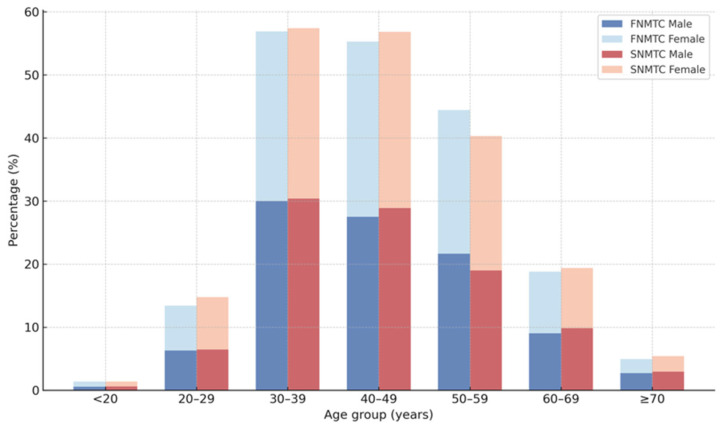
Age and sex distribution of patients with familial and sporadic NMTC (FNMTC vs. SNMTC).

Among the total 46,572 patients with NMTC, 3829 had a family history of NMTC, resulting in an FNMTC prevalence of 8.2% (3829/46,572). The overall age and sex distribution of patients with FNMTC and SNMTC is illustrated in Figure 2, and the detailed data are provided in Supplementary Table S1.

Patients with FNMTC were diagnosed at a significantly younger age than those with SNMTC (mean ± SD, 41.7 ± 12.6 vs. 44.2 ± 13.1 years; *p* < 0.001). When categorized by age, the proportion of younger patients (<30 years) was slightly lower in FNMTC compared with SNMTC (9.3% vs. 10.7%, *p* = 0.042), whereas the relative proportions of patients aged 30–59 years were comparable between the two groups. In contrast, elderly patients (≥70 years) were less frequent in the FNMTC group than in the SNMTC group (2.6% vs. 4.6%, *p* < 0.05). Female predominance was observed in both cohorts; however, the proportion of male patients was significantly higher among those with FNMTC than among those with SNMTC (22.8% vs. 19.3%, *p* < 0.001).

### 3.3. Comparison of Clinicopathological Characteristics of FNMTC and SNMTC

A comparative analysis was conducted to evaluate the clinicopathological characteristics of the patients with FNMTC and SNMTC; the results are shown in Table 1. The proportion of male patients was significantly higher in the FNMTC group than in the SNMTC group (22.8% vs. 19.3%, *p* < 0.001). The mean age did not differ significantly between groups (45.0 ± 12.1 vs. 44.7 ± 12.3 years, *p* = 0.068), and the age distribution was comparable (*p* = 0.3). Among the surgical approaches, robot-assisted surgery was slightly more common in the FNMTC group (23.7% vs. 22.7%), whereas open thyroidectomy remained the predominant method in both groups (*p* = 0.018). There were no significant differences in the extent of surgery between the groups (*p* = 0.2). In patients with FNMTC, tumor size was significantly smaller (0.9 ± 0.7 cm vs. 1.0 ± 0.9 cm, *p* < 0.001), with a higher proportion of microcarcinomas (≤10 mm) (71.8% vs. 68.0%, *p* < 0.001). However, FNMTC was associated with a higher frequency of bilaterality (23.5% vs. 17.5%, *p* < 0.001), multifocality (39.0% vs. 30.5%, *p* < 0.001), and extracapsular extension (53.7% vs. 51.1%, *p* = 0.002).

Histological subtype distribution showed a slightly higher proportion of papillary carcinomas in FNMTC (99.1% vs. 98.5%, *p* = 0.007), although aggressive histology did not differ significantly. Central lymph node (CN) metastasis was more frequent in the FNMTC group (41.5% vs. 38.8%, *p* = 0.001), whereas lateral lymph node (LLN) and distant metastasis rates were similar between groups.

The proportion of patients receiving RAIT was identical in both groups (34.9%), although high-dose RAIT was more common in FNMTC (13.5% vs. 12.3%, *p* = 0.035). There was no difference in the response to RAIT.

The recurrence rates were comparable between the two groups (1.9% in FNMTC vs. 2.3% in SNMTC; *p* = 0.1), and most recurrences were locoregional in both groups. Notably, the all-cause mortality rate was significantly lower in the FNMTC group (0.4% vs. 1.4%, *p* < 0.001), although thyroid cancer-specific mortality did not differ (*p* = 0.6). The mean follow-up duration was significantly shorter in FNMTC (44.6 ± 29.5 months) than in SNMTC (55.7 ± 42.2 months, *p* < 0.001).

Due to the complexity of analyzing a large heterogeneous cohort, patients were stratified according to ATA risk categories to facilitate more accurate and clinically relevant subgroup comparisons.

As seen in Table 2, among the 16,894 patients classified as low-risk based on the ATA guidelines, 1312 (7.8%) had FNMTC and 15,582 (92.2%) had sporadic NMTC (SNMTC). The baseline characteristics, including sex, age, and thyroid function, were comparable between the groups. The FNMTC group had significantly smaller tumors (*p* = 0.002) but showed higher rates of bilaterality (11.7% vs. 9.6%, *p* = 0.012) and multifocality (26.4% vs. 21.0%, *p* < 0.001). Central lymph node metastasis was slightly more frequent in FNMTC (9.1% vs. 7.7%, *p* = 0.067). The surgical extent and RAIT usage did not differ significantly. Recurrence rates were equally low (0.9%), and no distant metastases were observed in either group. Overall survival exceeded 99% in both groups, with no thyroid cancer-related deaths among patients with FNMTC. However, the FNMTC group had a significantly shorter follow-up duration (*p* < 0.001).

Among the 29,678 patients classified as intermediate-to-high-risk according to the ATA guidelines, 2517 (8.5%) had FNMTC and 27,161 (91.5%) had SNMTC. Patients with FNMTC were more likely to be male (25.1% vs. 20.5%, *p* < 0.001) and slightly older at diagnosis (*p* = 0.016). Tumors in patients with FNMTC were smaller on average (1.0 ± 0.8 cm vs. 1.2 ± 1.0 cm, *p* < 0.001) but showed significantly higher rates of bilaterality (29.6% vs. 22.1%, *p* < 0.001) and multifocality (45.6% vs. 35.9%, *p* < 0.001). High-dose RAI was slightly more frequent in FNMTC (20.4% vs. 19.1%, *p* = 0.05). The rates of extracapsular extension, central/lateral lymph node metastasis, and distant metastasis were similar between groups. Although the overall recurrence rate was slightly lower in FNMTC (2.3% vs. 3.0%, *p* = 0.051), this did not reach statistical significance. Most recurrences were locoregional. Importantly, overall survival was significantly higher in the FNMTC group (99.5% vs. 98.2%, *p* < 0.001), with only eight deaths in FNMTC patients, and only one attributed to thyroid cancer. The FNMTC group had a significantly shorter follow-up duration (45.6 ± 29.5 vs. 57.0 ± 42.8 months, *p* < 0.001), which may limit long-term outcome comparisons (Table 3).

We performed a subgroup analysis according to the affected family members and family member relationships of the patients with FNMTC. As seen in Table 4, among the 3829 patients with FNMTC, 3354 had one affected family member, 410 had two affected members, and 65 had three or more affected members. The proportion of male patients increased significantly as the number of affected family members increased (22.2% vs. 26.6% vs. 30.8%, *p* = 0.041), while the age distribution did not differ among groups. Mean tumor size was similar across groups, with more than 70% of cases being microcarcinomas. However, the incidence of bilaterality increased significantly as the number of affected family members increased (22.6% vs. 30.2% vs. 26.2%, *p* = 0.002), while multiplicity showed a non-significant increasing trend (*p* = 0.06). Rates of extracapsular extension, lymph node metastasis, and distant metastasis were comparable among the groups. The proportion of patients receiving radioactive iodine treatment (RAIT) was higher in families with two affected members (41.5%) compared with those with one (34.2%) or three or more (30.8%) (*p* = 0.001). Recurrence rates and survival outcomes showed no significant differences. The mean follow-up duration was approximately 45 months, and disease-specific mortality was extremely low (only one thyroid cancer-related death).

Among the 3829 patients with FNMTC, a subgroup analysis was performed based on familial relationships: 1762 patients had parent–offspring involvement, 1877 had sibling involvement, and 190 had a mixed pattern (both parent–offspring and siblings).

Sex distribution showed a significantly higher proportion of male patients in the mixed group (32.1%) than in the parent–offspring (23.1%) and sibling (21.6%) groups (*p* = 0.005). The mean age at diagnosis also differed significantly, with sibling-group patients being older (48.9 ± 10.4 years) than the parent–offspring (40.9 ± 12.7 years) and mixed (44.5 ± 10.4 years) groups (*p* = 0.001). Tumor size was similar among groups (overall mean 0.9 ± 0.7 cm), with a non-significant trend toward smaller tumors in the mixed group (*p* = 0.056). Extracapsular extension did not differ significantly. However, bilaterality was more prevalent in the sibling group (26.3%) than in the parent–offspring (20.3%) and mixed (24.7%) groups (*p* = 0.001). Central lymph node metastasis was significantly more frequent in the parent–offspring group (44.4%) than in the sibling (38.8%) and mixed (40.5%) groups (*p* = 0.003). Lateral neck node involvement and distant metastasis rates were low and comparable across all groups.

The use of RAIT varied significantly among the groups, with the highest rate in the sibling group (38.8%) and the lowest in the parent–offspring group (30.7%) (*p* = 0.001). The recurrence rate showed a non-significant trend, being higher in the mixed group (3.2%) than in the parent–offspring (2.2%) and sibling (1.4%) groups (*p* = 0.09). The mean follow-up duration differed slightly, being shortest in the parent–offspring group (42.4 ± 27.7 months) and longest in the sibling group (46.6 ± 30.8 months) (*p* = 0.007) (Table 5).

Only variables with statistically significant or clinically relevant differences are shown. Detailed numeric values and non-significant parameters are provided in Supplementary Tables S2–S5.

### 3.4. Recurrence-Free Survival Analysis of FNMTC and SNMTC

Patients who underwent surgery between 1990 and 2019 were selected for recurrence-free survival analysis (N = 32,976). Univariate and multivariate analyses were performed to identify factors associated with recurrence and determine the risk factors affecting disease-free survival (DFS).

Although overall survival was significantly higher in the FNMTC group, this difference may reflect non-cancer-related factors, such as younger age and fewer comorbidities, given the very low disease-specific mortality of differentiated thyroid carcinoma; hence, DFS provides the more clinically meaningful comparator between groups.

As seen in Table 6, a family history of thyroid cancer was independently associated with a significantly increased risk of recurrence (HR = 1.55, 95% CI: 1.16–1.91, *p* = 0.03). Among the surgical extent categories, bilateral total thyroidectomy was associated with a significantly reduced risk of recurrence compared with lobectomy (HR = 0.27, 95% CI: 0.13–0.56, *p* < 0.001). In contrast, lobectomy with partial or subtotal completion did not show a significant difference (HR = 0.68, *p* = 0.4). Tumor size was also a significant risk factor, with increasing size correlating with higher recurrence risk (HR = 1.41, 95% CI: 1.10–1.82, *p* = 0.008). The presence of extracapsular extension demonstrated a strong association with recurrence (HR = 4.46, 95% CI: 1.70–11.7, *p* = 0.002), as did central lymph node metastasis (HR = 6.73, 95% CI: 2.88–15.7, *p* < 0.001), indicating that these are potent independent predictors of poor DFS. Other variables, including sex, age, bilaterality, multiplicity, and lateral lymph node metastasis, were not statistically significant in the multivariate model.

To evaluate the recurrence risk according to the ATA risk category, multivariate Cox proportional competing risk analyses were performed separately in the low- and intermediate-to-high-risk groups (Table 7 and Table 8). In the low-risk group (Table 7), total thyroidectomy was independently associated with a significantly lower risk of recurrence compared with lobectomy (HR = 0.25, *p* = 0.001). Lymph node metastasis was a strong predictor of recurrence (HR = 12.1, *p* = 0.001), whereas other factors—including family history, sex, age, bilaterality, and multiplicity—did not reach statistical significance.

Several variables were independently associated with recurrence in the intermediate-to-high-risk group (Table 8). Family history (HR = 1.65, *p* < 0.001), extracapsular extension (HR = 1.47, *p* < 0.001), multiplicity (HR = 1.30, *p* = 0.02), and lymph node metastasis (HR = 2.95, *p* < 0.001) were significant risk factors. Female sex was associated with a lower risk (HR = 0.60, *p* < 0.001), and both subtotal and total thyroidectomies were more protective compared with lobectomies.

These findings suggest that the impact of certain risk factors, such as family history and lymph node metastasis, may differ in strength and significance depending on the underlying risk category, highlighting the importance of risk-adapted recurrence surveillance.

**Figure 3:**
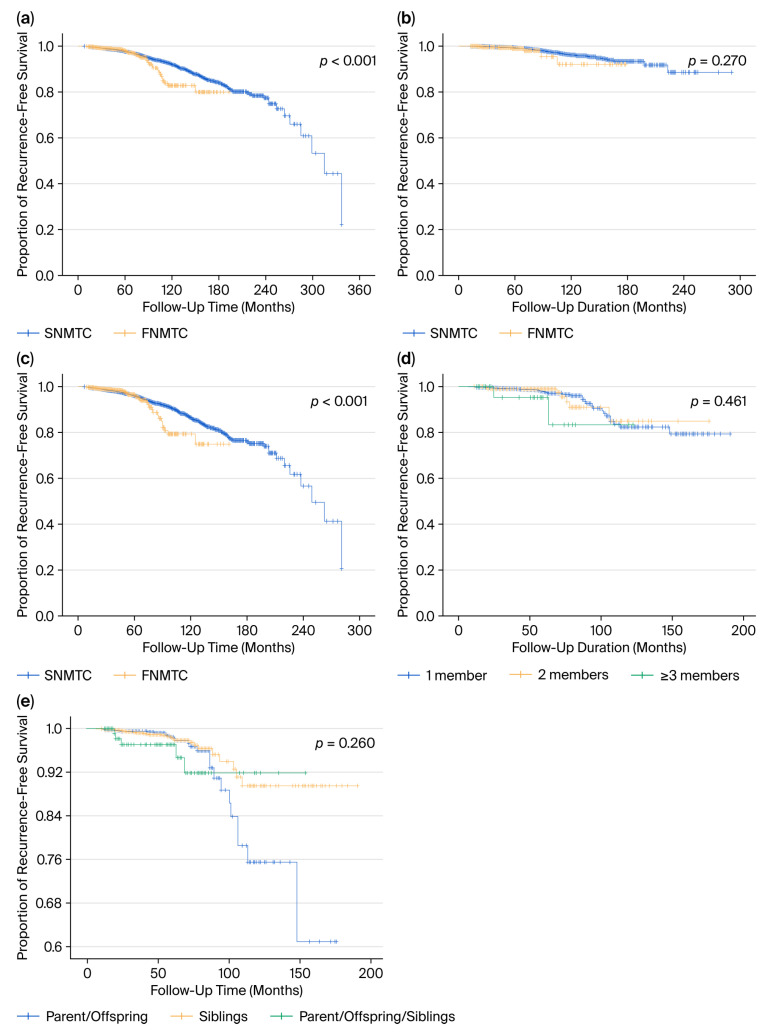
(**a**) Recurrence-free survival curves of FNMTC and SNMTC. (**b**) Recurrence-free survival curves of the low-risk group. (**c**) Recurrence-free survival curves of the intermediate–high-risk group. (**d**) Recurrence-free survival curves based on parent/offspring, sibling, and parent/offspring/sibling FNMTC hereditary forms. (**e**) Recurrence-free survival curves based on affected family members of patients with FNMTC.

Figure 3 illustrates Kaplan–Meier recurrence-free survival (RFS) curves comparing familial and sporadic non-medullary thyroid carcinoma (FNMTC vs. SNMTC) and stratified subgroups.

## 4. Discussion

With the increasing incidence of thyroid cancer, the number of patients with FNMTC has also increased. The present study showed that FNMTC accounted for a significant proportion of NMTC cases, with a prevalence of 8.2% in the total NMTC cohort. The reported prevalence of FNMTC is 3–9% of all thyroid cancers [9,10,25]. Several large population-based studies have indicated that patients’ relatives have a higher risk of developing the same type of cancer. In some cohorts, the risk of thyroid cancer has been reported to be the highest among all cancer types. An FNMTC diagnosis is made if two or more first-degree relatives develop NMTC without other known associated cancers [26]. Specifically, FNMTC is defined as differentiated thyroid cancer that occurs in at least two first-degree relatives, including the index patient, without other predisposing causes of thyroid cancer [9]. However, this definition is controversial, because if only two relatives are affected, there is a 62% to 66% probability that the two tumors are sporadic [13]. On the other hand, families with three or more affected members are rare, accounting for less than 5% of the major FNMTC series. In this study, patients were classified as having FNMTC if they had at least one first-degree relative with the disease. The prevalence of patients with two affected first-degree relatives, including the patient, was 7.31%, while the prevalence of those with three or more affected first-degree relatives was 0.8%.

As reported by Park et al. and others, the frequency of thyroid cancer diagnosis in Korea increased sharply in the early 2000s. However, after the debate on the overdiagnosis of thyroid cancer, which was highlighted in the New England Journal of Medicine, the number of diagnoses temporarily declined. Since the late 2010s, its incidence has steadily increased. The number of thyroid cancer surgeries performed at our institution showed a similar trend. As the number of thyroid cancer diagnoses has increased, the proportion of patients with FNMTC has also increased [2,27].

Over the course of the study period (1990–2024), diagnostic and clinical practices had evolved substantially. In particular, the introduction and widespread use of high-resolution neck ultrasonography have markedly enhanced the accuracy and sensitivity of thyroid cancer detection, enabling the identification of smaller and subclinical tumors that might have gone unnoticed in earlier decades. Improvements in cytological techniques and guideline-based management strategies have further contributed to earlier diagnosis and more tailored surgical planning. Although these advancements have influenced the overall detection rate and tumor characteristics over time, both familial and sporadic cases were subject to the same diagnostic environment within each time frame, and thus the relative comparisons between the groups remain valid. The consistent trends observed across subgroups in our large cohort suggest that the key findings of this study reflect inherent biological differences rather than temporal diagnostic variations.

Our study also highlighted notable sex differences in the prevalence and inheritance of FNMTC. This disease is more common in women, which is consistent with the well-documented higher incidence of thyroid cancer in women. The proportion of FNMTC cases has also increased over time, particularly after 2014, although the total number of surgeries has decreased. This trend suggests that familial thyroid cancer cases may have remained relatively stable or even increased in incidence, highlighting the need for enhanced screening of at-risk individuals. In our cohort, FNMTC demonstrated a peak prevalence in the 35–59-year age range, with a younger mean age at diagnosis compared with SNMTC. This finding suggests that familial predisposition may contribute to an earlier disease onset, consistent with previous reports.

Interestingly, although female predominance is a well-recognized feature of non-medullary thyroid carcinoma, our study showed that the proportion of male patients modestly increased as the number of affected family members rose. The biological or environmental basis of this observation remains uncertain. This pattern may reflect subtle sex-related differences in disease susceptibility or expression within families with stronger aggregation, but such an interpretation should be made cautiously. Alternatively, sociodemographic or behavioral factors, including differential participation in screening or family-based surveillance, may also contribute to this trend. Previous studies have reported inconsistent findings regarding sex-related differences in familial thyroid cancer. Some series suggested that male patients may present with more advanced disease or higher recurrence risk compared with females [21,24]. In contrast, others found no significant difference after adjusting for age and tumor stage [13,28]. Therefore, further research integrating genetic, hormonal, and behavioral determinants is warranted to clarify whether this shift in gender distribution has any impact on disease presentation or outcomes in FNMTC.

The biological characteristics of the disease, including the prognosis of patients with a family history of DTC, remain controversial. Most studies have reported more aggressive disease at presentation with worse outcomes in the FNMTC group [14,15]. However, several studies have reported more aggressive disease at presentation with similar outcomes at the end of follow-up or identical baseline characteristics with similar or worse outcomes [16,17,18,20,25]. Studies recommending extensive treatment for patients with a family history of DTC have reported no difference in prognosis between patients with FNMTC and those with SNMTC [29,30]. While numerous studies on familial DTC have been conducted, most have included heterogeneous patient cohorts with significantly smaller familial groups. The makeup of these cohorts may have imposed several limitations on the linear analysis. This study was conducted on a large patient population and is meaningful in that it not only compared aggressiveness at the time of diagnosis, but also followed up on recurrence. Interestingly, although the FNMTC group exhibited smaller tumor sizes on average, it showed significantly higher frequencies of bilaterality, multifocality, and central lymph node metastasis compared with the SNMTC group. This counterintuitive pattern suggests that familial predisposition may contribute to distinct tumorigenic pathways leading to more extensive local disease despite smaller primary lesions.

Previous studies have reported aggressiveness and outcomes based on the number of affected family members, and some have recommended more invasive surgery for patients with three or more first-degree relatives [14,15,17,24]. When FNMTC cases were categorized based on the number of affected family members, it was observed that the rates of bilaterality and multiplicity decreased as the number of affected family members increased.

To enhance the clinical relevance of our findings and reduce the potential heterogeneity inherent in large cohort studies, we stratified patients with FNMTC and SNMTC according to the ATA risk stratification system [29]. This approach allowed us to contextualize clinicopathological differences within clinically meaningful prognostic categories and examine whether family history exerted a differential influence depending on baseline recurrence risk. Risk stratification provides a more nuanced understanding of the behavior and outcomes of thyroid cancer. In the low-risk group, both FNMTC and SNMTC patients demonstrated excellent prognoses, with extremely low recurrence rates and no thyroid cancer-specific mortality. However, the significantly higher rates of bilaterality and multifocality in patients with FNMTC suggest that familial predisposition may contribute to more extensive local disease, independent of traditional prognostic indicators. In contrast, among intermediate-to-high-risk patients, FNMTC was found to be associated with higher bilaterality and multifocality. Despite these locally invasive features, the tumor size is paradoxically smaller in patients with FNMTC. This observation may reflect an earlier diagnosis due to increased surveillance in families with a known history of thyroid cancer, or alternatively, inherent differences in tumor growth dynamics. Extracapsular extension and central lymph node metastasis were also more common in patients with FNMTC in the overall cohort, although lateral lymph node and distant metastasis rates were comparable between the groups. These results suggest that while FNMTC may present with more extensive local disease, the propensity for distant spread is similar to that of sporadic tumors.

There are three hereditary forms of FNMTC (parent/offspring, sibling, and parent/offspring/sibling), each with unique clinical characteristics. Park et al. reported that parent/offspring FNMTC exhibited more frequent extrathyroidal invasion and a higher recurrence rate than SNMTC in a classic study based on a large sample size. In contrast, sibling FNMTC exhibited a higher prevalence in women, smaller tumor size, and a higher incidence of Hashimoto’s thyroiditis than SNMTC [14]. Moreover, Cao et al. reported that despite an earlier disease onset in the parent/offspring group, there were no other significant differences in the clinicopathological and outcome characteristics between the three hereditary forms of FNMTC [15]. In this study, we divided patients with FNMTC according to their hereditary forms (parent/offspring, sibling, and parent/offspring/sibling types). There were no significant differences in the factors related to tumor aggressiveness. However, the sibling group had the highest average age, and the parent/offspring/sibling group had the highest proportion of male patients.

There seems to be a lack of consensus concerning the impact of the family history of PTC on DFS. In this study, the recurrence-free survival analysis revealed that the family history of thyroid cancer was independently associated with an increased risk of recurrence across the entire cohort. This finding aligns with those of prior studies suggesting that familial tumors often exhibit multifocality, bilaterality, and a higher rate of local invasiveness, which may predispose them to recurrence even after initial treatment. When stratifying according to ATA risk categories, the impact of family history was more pronounced in the intermediate-to-high-risk group (HR =1.65, *p* < 0.001) but was not statistically significant in the low-risk group (HR = 1.94, *p* = 0.075). This suggests that familial predisposition has a greater influence on disease progression in the presence of other aggressive features. Consequently, patients with FNMTC in higher-risk strata may benefit from closer surveillance and more aggressive initial management strategies. Extracapsular extension, central lymph node metastasis, and multiplicity were identified as independent predictors of recurrence in both the general and intermediate-to-high-risk populations, consistent with the established prognostic models. Notably, lymph node metastasis had an exceptionally strong association with recurrence in low-risk patients (HR = 12.1, *p* = 0.0001), indicating that even in this otherwise favorable subgroup, the presence of lymph node metastasis warrants careful postoperative follow-up. Although recurrence-free survival provides a reliable measure of disease-specific prognosis, overall survival (OS) should be interpreted with caution in differentiated thyroid carcinoma, as disease-specific mortality is exceedingly low. Most deaths in this population are attributable to non-cancer-related causes, particularly in older patients with comorbidities. Therefore, the slightly higher OS observed in the FNMTC group in our cohort is more likely explained by favorable baseline characteristics—such as younger age at diagnosis, smaller tumor size, and better general health status—rather than by intrinsic differences in tumor biology or treatment response. In this context, DFS offers a more clinically meaningful indicator of disease behavior and long-term prognosis than OS in both familial and sporadic NMTC. Moreover, the shorter follow-up in the FNMTC group may have further limited the accrual of non-cancer deaths, inflating OS relative to SNMTC.

Our results also revealed that certain risk factors exerted different influences depending on the baseline ATA risk category. While family history and multiplicity were significant in the intermediate-to-high-risk group, they did not reach significance in the low-risk group. This finding highlights the potential role of family history as a supplementary factor in risk-adapted recurrence surveillance, supporting the need for more individualized follow-up strategies in patients with FNMTC, particularly those in intermediate-to-high-risk categories. The results suggest that incorporating familial predisposition into clinical decision-making may help optimize the surveillance intensity and early detection of recurrence. Future prospective studies are warranted to evaluate whether integrating family history into risk stratification models can lead to improved patient outcomes.

Nevertheless, these findings should be interpreted in light of several limitations. First, the definition of familial non-medullary thyroid carcinoma (FNMTC) in our study was based on the presence of at least one first-degree relative, other than the index patient, diagnosed with non-medullary thyroid carcinoma. Although this operational definition has been adopted in several epidemiologic studies, it may not fully differentiate true hereditary cases from coincidental familial clustering, particularly in regions where thyroid cancer is highly prevalent. As such, our results may reflect a mixture of genetically predisposed and environmentally influenced familial cases.

Second, information on family history was collected through patient self-reports obtained at the initial outpatient visit or upon hospital admission, without pathological confirmation of the relatives’ diagnoses. This reliance on patient recall may have introduced recall bias or misclassification regarding familial status. However, we believe the potential impact of this limitation on the main findings is likely modest, as family history was systematically assessed at the time of diagnosis using a standardized approach, and the large sample size and consistent trends across subgroups suggest that any random misclassification would not substantially alter the observed associations. Still, the proportion of FNMTC may have been either underestimated or overestimated, and this should be considered when interpreting the results.

Third, because the analysis was performed using data from probands only, this study could not explore intrafamilial heterogeneity or genotype–phenotype correlations among affected relatives. Moreover, systematic screening of unaffected family members was not evaluated, limiting the assessment of potential detection bias.

Fourth, although the overall follow-up duration was shorter in the FNMTC group than in the sporadic NMTC group, which may have led to a potential underestimation of late recurrences, the recurrence-free survival trends observed in our study are supported by consistent findings in the adjusted analyses. Multivariable Cox regression incorporating major prognostic variables confirmed that family history was independently associated with recurrence, particularly in the intermediate-to-high-risk category.

Future prospective studies incorporating verified family histories, pathological confirmation, and genetic testing across multiple affected members are warranted to validate these findings and further elucidate the hereditary features of FNMTC.

Despite these limitations, the large-scale, nationally representative cohort and comprehensive clinicopathological data in this study provide valuable and robust insights into the phenotype and clinical behavior of familial non-medullary thyroid carcinoma in a real-world population.

## 5. Conclusions

This large cohort study demonstrated that familial non-medullary thyroid carcinoma (FNMTC) constitutes a notable subset of NMTC and exhibits distinct clinicopathological characteristics, including higher multifocality, bilaterality, and central lymph node metastasis, despite smaller tumor size. Family history was identified as an independent predictor of recurrence, particularly in the intermediate-to-high ATA risk group, whereas its impact was minimal in low-risk patients. These findings suggest that FNMTC represents a biologically heterogeneous entity, and patients with FNMTC may benefit from risk-adapted management strategies, with closer surveillance warranted for high-risk familial cases. While our results underscore the prognostic significance of family history, this study was not designed to propose direct modifications to current treatment guidelines. Instead, family history may serve as a supplementary factor in refining risk stratification and follow-up intensity. Future prospective, multi-center studies incorporating genetic profiling and validated familial data are needed to clarify the hereditary basis of FNMTC and determine whether integrating family history into clinical decision-making can improve patient outcomes.

## Figures and Tables

**Table 1:** Clinicopathological characteristics, treatment modalities, and outcomes of SNMTC versus FNMTC.

	Overall N = 46,572	SNMTC N = 42,743	FNMTC N = 3829	*p*-Value
Female, n (%)	37,448 (80.4)	34,492 (80.7)	2956 (77.2)	
Age, years (mean ± SD)	44.7 ± 12.3	44.7 ± 12.3	45.0 ± 12.1	0.068
Thyroid function abnormal, n (%)				0.2
Hypothyroidism	636 (1.4)	575 (1.4)	61 (1.6)	
Hyperthyroidism	303 (0.7)	284 (0.7)	19 (0.5)	
Operation method, n (%)				0.018
Open	28,709(61.6)	26,392 (61.7)	2317 (60.5)	
Minimal incision	6342 (13.6)	5792 (13.6)	550 (14.4)	
Endoscopic	889 (1.9)	836 (2.0)	53 (1.4)	
Robot	10,632 (22.8)	9723 (22.7)	909 (23.7)	
Operation name, n (%)				0.2
Lobectomy	20,889 (44.9)	19,197 (44.9)	1692 (44.2)	
Lobectomy + partial or subtotal	5871 (12.6)	5413 (12.7)	458 (12.0)	
Bilateral total	19,812(42.5)	18,133 (42.4)	1679 (43.8)	
Tumor size, cm (mean ± SD)	1.0 ± 0.9	1.0 ± 0.9	0.9 ± 0.7	<0.001
Tumor size group, n (%)				<0.001
≤10 mm	31,797 (68.3)	29,048 (68.0)	2749 (71.8)	
10~20 mm	10,829 (23.3)	9962 (23.3)	867 (22.6)	
20~40 mm	3255 (7.0)	3075 (7.2)	180 (4.7)	
>40 mm	691 (1.5)	658 (1.5)	33 (0.9)	
Bilaterality, n (%)	8385 (18.0)	7486 (17.5)	899 (23.5)	<0.001
Multiplicity, n (%)	14,515 (31.2)	13,020 (30.5)	1495 (39.0)	<0.001
Extracapsular extension, n (%)	23,879 (51.3)	21,824 (51.1)	2055 (53.7)	0.002
Pathology result, n (%)				0.007
Papillary ca.	45,888 (98.5)	42,095 (98.5)	3793 (99.1)	
Follicular ca.	581 (1.2)	547 (1.3)	34 (0.9)	
Oncocytic ca.	103 (0.2)	101 (0.2)	2 (0.1)	
Aggressive pathology *	420 (0.9)	390 (0.9)	30 (0.8)	0.4
CLN ^†^ metastasis, n (%)	18,171 (39.0)	16,583 (38.8)	1588 (41.5)	0.001
LLN ^‡^ metastasis, n (%)	4482 (9.6)	4119 (9.6)	363 (9.5)	0.8
Distant metastasis, n (%)				0.7
None	46,372 (99.6)	42,558 (99.6)	3814 (99.6)	
Synchronous	139 (0.3)	130 (0.3)	9 (0.2)	
Metachronous	61 (0.1)	55 (0.1)	6 (0.2)	
Distant metastasis organ, n (%)				0.5
Lung	169 (82.4)	157 (83.1)	12 (75.0)	
Bone	25 (12.2)	22 (11.6)	3 (18.8)	
Brain	3 (1.5)	3 (1.6)	0 (0.0)	
Multiple	5 (2.4)	4 (2.1)	1 (6.3)	
Other	3 (1.5)	3 (1.6)	0 (0.0)	
RAIT ^§^, n (%)	16,233 (34.9)	14,897 (34.9)	1336 (34.9)	>0.9
RAIT dose, n (%)				0.035
Low dose	10,515 (22.8)	9693 (22.9)	822 (21.6)	
High dose	5718 (12.4)	5204 (12.3)	514 (13.5)	
RAIT result, n (%)				0.3
No or minimal uptake	16,151 (99.4)	14,823 (99.5)	1328 (99.3)	
Hot uptake	91 (0.6)	81 (0.5)	10 (0.7)	
Recurrence, n (%)	1038 (2.2)	967 (2.3)	71 (1.9)	0.1
Recurrence site, n (%)				0.6
Local	953 (91.8)	887 (91.7)	66 (93.0)	
Distant	51 (4.9)	48 (5.0)	3 (4.2)	
Local + distant	34 (3.3)	32 (3.3)	2 (2.8)	
Survival, n (%)				<0.001
Alive	30,605(98.7)	28,080 (98.6)	2525 (99.6)	
Death	413 (1.3)	402 (1.4)	11 (0.4)	
Cause of death, n (%)				0.6
Thyroid cancer	90 (21.3)	89 (21.6)	1 (9.1)	
Other cause	333 (78.7)	323 (78.4)	10 (90.9)	
Follow-up duration, months (mean ± SD)	54.7 ± 41.4	55.7 ± 42.2	44.6 ± 29.5	<0.001

Aggressive pathology *: Hobnail, tall cell, columnar cell, diffuse sclerosing variant. CLN ^†^: Central lymph node. LLN ^‡^: Lateral lymph node. RAIT ^§^: Radioactive iodine treatment.

**Table 2:** Clinicopathologic characteristics, treatment modalities, and outcomes of SNMTC versus FNMTC in the low-risk group.

Characteristics	Overall N = 16,894	SNMTC N = 15,582	FNMTC N = 1312	*p*-Value
Female, n (%)	13,975 (82.7)	12,905 (82.8)	1070 (81.6)	0.2
Age, years (mean ± SD)	45.1 ± 11.6	45.1 ± 11.6	45.0 ± 11.5	0.9
Tumor size, cm (mean ± SD)	0.7 ± 0.6	0.7 ± 0.6	0.7 ± 0.5	0.002
Bilaterality, n (%)	1650 (9.8)	1496 (9.6)	154 (11.7)	0.012
Multiplicity, n (%)	3611 (21.4)	3265 (21.0)	346 (26.4)	<0.001
CLN * metastasis, n (%)	1325 (7.8)	1205 (7.7)	120 (9.1)	0.067
RAIT ^†^, n (%)	2018 (11.9)	1872 (12.0)	146 (11.1)	0.3
Recurrence, n (%)	155 (0.9)	143 (0.9)	12 (0.9)	>0.9
Survival, n (%)				0.2
Alive	10,747 (99.3)	9929 (99.3)	818 (99.6)	
Death	78 (0.7)	75 (0.7)	3 (0.4)	
Cause of death, n (%)				>0.9
Thyroid cancer	9 (12.2)	9 (12.5)	0	
Other cause	65 (87.9)	63 (87.5)	2 (100.0)	
Follow-up duration, months (mean ± SD)	52.4 ± 40.3	53.2 ± 41.0	42.6 ± 29.3	<0.001

CLN *: Central lymph node. RAIT ^†^: Radioactive iodine treatment.

**Table 3:** Clinicopathologic characteristics, treatment modalities, and outcomes of SNMTC versus FNMTC in the intermediate–high-risk group.

Characteristics	Overall N = 29,678	SNMTC N = 27,161	FNMTC N = 2517	*p*-Value
Female, n (%)	23,473 (79.1)	21,587 (79.5)	1886 (74.9)	<0.001
Age, years (mean ± SD)	44.5 ± 12.6	44.4 ± 12.6	45.0 ± 12.4	0.016
Tumor size, cm (mean ± SD)	1.2 ± 0.9	1.2 ± 1.0	1.0 ± 0.8	<0.001
Bilaterality, n (%)	6735 (22.7)	5990 (22.1)	745 (29.6)	<0.001
Multiplicity, n (%)	10,904 (36.7)	9755 (35.9)	1149 (45.6)	<0.001
Extracapsular extension, n (%)	23,879 (80.5)	21,824 (80.4)	2055 (81.6)	0.12
CLN ^†^ metastasis, n (%)	16,846 (56.8)	15,378 (56.6)	1468 (58.3)	0.1
LLN ^‡^ metastasis, n (%)	4482 (15.1)	4119 (15.2)	363 (14.4)	0.3
Distant metastasis, n (%)				0.6
None	29,478 (99.3)	26,976 (99.3)	2502 (99.4)	
Synchronous	139 (0.5)	130 (0.5)	9 (0.4)	
Metachronous	61 (0.2)	55 (0.2)	6 (0.2)	
RAIT ^§^, n (%)	14,215 (47.9)	13,025 (48.0)	1190 (47.3)	0.5
Recurrence, n (%)	883 (3.0)	824 (3.0)	59 (2.3)	0.051
Survival, n (%)				<0.001
Alive	19,858 (98.3)	18,151 (98.2)	1707 (99.5)	
Death	335 (1.7)	327 (1.8)	8 (0.5)	
Cause of death, n (%)				0.5
Thyroid cancer	81 (23.2)	80 (23.5)	1 (11.1)	
Other cause	268 (76.8)	260(76.5)	8 (88.6)	
Follow-up duration, months (mean ± SD)	56.0 ± 41.9	57.0 ± 42.8	45.6 ± 29.5	<0.001

CLN ^†^: Central lymph node. LLN ^‡^: Lateral lymph node. RAIT ^§^: Radioactive iodine treatment.

**Table 4:** Clinicopathological characteristics, treatment modalities, and outcomes of FNMTC based on the affected family members.

Characteristics	Overall N = 3829	One Affected N = 3354	Two Affected N = 410	Three or More Affected N = 65	*p*-Value
Female, n (%)	2956 (77.2)	2610 (77.8)	301 (73.4)	45 (69.2)	0.041
Age, years (mean ± SD)	45.0 ± 12.1	45.0 ± 12.2	44.9 ± 11.3	43.8 ± 11.4	0.8
Tumor size, cm (mean ± SD)	0.9 ± 0.7	0.9 ± 0.7	0.9 ± 0.6	0.8 ± 0.6	0.087
Bilaterality, n (%)	899 (23.5)	758 (22.6)	124 (30.2)	17 (26.2)	0.002
Multiplicity, n (%)	1495 (39.0)	1286 (38.3)	181 (44.1)	28 (43.1)	0.06
Extracapsular extension, n (%)	2055 (53.7)	1800 (53.7)	222 (54.1)	33 (50.8)	0.9
CLN ^†^ metastasis, n (%)	1588 (41.5)	1380 (41.1)	181 (44.1)	27 (41.5)	0.5
LLN ^‡^ metastasis, n (%)	363 (9.5)	314 (9.4)	44 (10.7)	5 (7.7)	0.6
Distant metastasis, n (%)					>0.9
None	3814 (99.6)	3340 (99.6)	409 (99.8)	65 (100.0)	
Synchronous	9 (0.2)	8 (0.2)	1 (0.2)	0	
Metachronous	6 (0.2)	6 (0.2)	0	0	
RAIT ^§^, n (%)	1336 (34.9)	1146 (34.2)	170 (41.5)	20 (30.8)	0.011
Recurrence, n (%)	71 (1.9)	57 (1.7)	12 (2.7)	2 (3.1)	0.2
Follow-up duration, months (mean ± SD)	44.6 ± 29.5	44.3 ± 29.3	47.5 ± 31.1	41.7 ± 28.6	0.2

CLN ^†^: Central lymph node. LLN ^‡^: Lateral lymph node. RAIT ^§^: Radioactive iodine treatment.

**Table 5:** Clinicopathological characteristics, treatment modalities, and outcomes of FNMTC based on hereditary forms.

	Overall N = 3829	Parent/Offspring N = 1762	Sibling N = 1877	Parent/Offspring/Sibling N = 190	*p*-Value
Female, n (%)	2956 (77.2)	1355 (76.9)	1472 (78.4)	129 (67.9)	0.005
Age, years (mean ± SD)	45.0 ± 12.1	40.9 ± 12.7	48.9 ± 10.4	44.5 ± 10.4	0.001
Tumor size, cm (mean ± SD)	0.9 ± 0.7	0.9 ± 0.7	0.9 ± 0.7	0.8 ± 0.6	0.056
Bilaterality, n (%)	899 (23.5)	358 (20.3)	494 (26.3)	47 (24.7)	0.001
Multiplicity, n (%)	1495 (39.0)	655 (37.2)	765 (40.8)	75 (39.5)	0.085
Extracapsular extension, n (%)	2055 (53.7)	921 (52.3)	1041 (55.5)	93 (48.9)	0.063
CLN ^†^ metastasis, n (%)	1588 (41.5)	783 (44.4)	728 (38.8)	77 (40.5)	0.003
LLN ^‡^ metastasis, n (%)	363 (9.5)	168 (9.5)	183 (9.7)	12 (6.3)	0.3
Distant metastasis, n (%)					>0.9
None	3814 (99.6)	1756 (99.7)	1868 (99.5)	190 (100.0)	
Synchronous	9 (0.2)	4 (0.2)	5 (0.3)	0	
Metachronous	6 (0.2)	2 (0.1)	4 (0.2)	0	
RAIT ^§^, n (%)	1336 (34.9)	541 (30.7)	729 (38.8)	66 (34.7)	0.001
Recurrence, n (%)	71 (1.9)	38 (2.2)	27 (1.4)	6 (3.2)	0.09
Follow-up duration, months (mean ± SD)	44.6 ± 29.5	42.4 ± 27.7	46.6 ± 30.8	44.7 ± 30.5	0.007

CLN ^†^: Central lymph node. LLN ^‡^: Lateral lymph node. RAIT ^§^: Radioactive iodine treatment.

**Table 6:** Cox proportional multivariate risk analysis of the clinical and pathological variables for disease-free survival in all patients.

Total Patients (N = 32,976)	HR	*p*-Value	95% CI	
Family history	1.55	0.03	1.16	1.91
Sex (female)	1.12	0.7	0.58	2.15
Age	0.47	0.12	0.19	1.21
Op range				
Lobectomy	Ref.			
Lobectomy + partial or subtotal	0.68	0.4	0.27	1.73
Bilateral total	0.27	<0.001	0.13	0.56
Tumor size	1.41	0.008	1.10	1.82
Extracapsular extension	4.46	0.002	1.70	11.7
Bilaterality	0.68	0.5	0.25	1.85
Multiplicity	1.14	0.8	0.51	2.55
CLN * metastasis	6.73	<0.001	2.88	15.7
LLN ^†^ metastasis	1.69	0.2	0.79	3.58

CLN *: Central lymph node. LLN ^†^: Lateral lymph node.

**Table 7:** Cox proportional multivariable competing risk analysis of the clinical and pathological variables for disease-free survival in the low-risk group.

Low-Risk Patients (N = 11,416)	HR	*p*-Value	95% CI	
Family history	1.94	0.075	0.93	4.03
Sex (female)	0.77	0.3	0.46	1.31
Age	1.08	0.8	0.66	1.77
Op range				
Lobectomy	Ref.			
Lobectomy + partial or subtotal	0.8	0.3	0.54	1.19
Bilateral total	0.25	0.001	0.14	0.42
Bilaterality	0.81	0.6	0.34	1.94
Multiplicity	1.38	0.2	0.83	2.32
Lymph node metastasis	12.1	0.001	4.62	31.5

**Table 8:** Cox proportional multivariable competing risk analysis of clinical and pathological variables for disease-free survival in the intermediate–high-risk group.

Intermediate–High-Risk Patients (N = 21,560)	HR	*p*-Value	95% CI	
Family history	1.65	<0.001	1.23	2.21
Sex (female)	0.6	<0.001	0.51	0.71
Age	1.08	0.8	0.66	1.77
Op range				
Lobectomy	Ref.			
Lobectomy + partial or subtotal	0.66	0.002	0.50	0.86
Bilateral total	0.52	<0.001	0.42	0.63
Extracapsular extension	1.47	<0.001	1.22	1.77
Bilaterality	1.26	0.06	0.99	1.61
Multiplicity	1.3	0.02	1.04	1.63
Lymph node metastasis	2.95	<0.001	2.43	3.58

## Data Availability

The original contributions presented in this study are included in this article; further inquiries can be directed to the corresponding author.

## Supplementary Materials

The following supporting information can be downloaded at: https://www.mdpi.com/article/10.3390/cancers17203381/s1, Table S1: Age and sex distribution of familial and sporadic non-medullary thyroid cancer; Table S2: Clinicopathologic characteristics, treatment modalities, and outcomes of SNMTC versus FNMTC in the low-risk group; Table S3: Clinicopathologic characteristics, treatment modalities, and outcomes of SNMTC versus FNMTC in the intermediate–high-risk group; Table S4: Clinicopathological characteristics, treatment modalities, and outcomes of FNMTC based on the affected family members; Table S5: Clinicopathological characteristics, treatment modalities, and outcomes of FNMTC based on hereditary forms.

## Abbreviations

FNMTC	Familial non-medullary thyroid cancer
NMTC	Non-medullary thyroid cancer
SNMTC	Sporadic non-medullary thyroid cancer
