# Supplementary material for: Familial Non-Medullary Thyroid Carcinoma: Distinct Clinicopathological Features and Prognostic Implications in a Large Cohort of 46,572 Patients
Source: Cancers (Basel). 2025 Oct 20;17(20):3381. doi: 10.3390/cancers17203381 (PMC12562981; doi:10.3390/cancers17203381)
Supplement: Supplementary file 1 [file cancers-17-03381-s001.zip › cancers-3910975-supplementary.pdf]

Supplementary Materials

Table S1. Age and sex distribution of familial and sporadic non-medullary thyroid cancer.

	FNMTC						SNMTC					
	Total		Male		Female		Total		Male		Female	
Age	No.	%	No.	%	No.	%	No.	%	No.	%	No.	%
5~9	1	0.03	0	0	1	0.03	8	0.02	4	0.05	4	0.01
10~14	4	0.1	0	0	4	0.14	45	0.11	14	0.17	31	0.09
15~19	24	0.63	5	0.57	19	0.64	268	0.63	31	0.38	237	0.69
20~24	97	2.53	19	2.18	78	2.64	1068	2.5	159	1.93	909	2.64
25~29	265	6.92	55	6.3	210	7.1	3402	7.96	531	6.44	2871	8.32
30~34	473	12.35	101	11.57	372	12.58	5480	12.82	1130	13.7	4350	12.61
35~39	584	15.25	161	18.44	423	14.31	6339	14.83	1377	16.69	4962	14.39
40~44	570	14.88	136	15.58	434	14.68	6459	15.11	1321	16.01	5138	14.9
45~49	490	12.82	103	11.91	387	13.09	5557	13	1061	12.86	4496	13.04
50~54	481	12.56	101	11.57	380	12.85	5056	11.83	850	10.3	4206	12.19
55~59	382	9.97	88	10.08	294	9.94	3871	9.06	718	8.7	3153	9.14
60~64	253	6.61	49	5.61	204	6.9	2654	6.21	506	6.13	2148	6.23
65~69	115	3	30	3.44	85	2.87	1442	3.37	307	3.72	1135	3.29
70~74	54	1.41	12	1.37	42	1.42	742	1.74	162	1.96	580	1.68
75~79	32	0.84	10	1.15	22	0.74	268	0.63	59	0.72	209	0.61
80~84	3	0.08	1	0.11	2	0.07	61	0.14	17	0.21	44	0.13
85~89	1	0.03	1	0.11	0	0	13	0.03	3	0.04	10	0.03
>=90	0	0	0	0	0	0	9	0.02	1	0.01	8	0.02
Total	3,829	100	872	100	2957	100	42,742	100	8,251	100	34,491	100

Table S2. Clinicopathologic characteristics, treatment modalities, and outcomes of SNMTC versus FNMTC in the low-risk group.

**Characteristics**	**Overall N = 16,894**	**SNMTC N = 15,582**	**FNMTC N = 1,312**	**p-value**
Sex, n(%)				0.2
Male	2,919 (17.3)	2,677 (17.2)	242 (18.4)	
Female	13,975 (82.7)	12,905 (82.8)	1,070 (81.6)	
Age, years (mean±SD)	45.1 ± 11.6	45.1 ± 11.6	45.0 ± 11.5	0.9
Age (group), n(%)				0.8
< 55 years	13,358 (79.1)	12,317 (79.0)	1,041 (79.3)	
≥ 55 years	3,536 (20.9)	3,265 (21.0)	271 (20.7)	
Operation method, n(%)				
Open	9,322 (55.2)	8,613 (55.3)	709 (54.0)	
Minimal incision	3,041 (18.0)	2,795 (17.9)	246 (18.8)	
Endoscopic	431 (2.6)	406 (2.6)	25 (1.9)	
Robot	4,100 (24.3)	3,768 (24.2)	332 (25.3)	
Operation name, n(%)				0.5
Lobectomy	10,072 (59.6)	9,290 (59.6)	782 (59.6)	
Lobectomy + partial or subtotal	2,748 (16.3)	2,548 (16.4)	200 (15.2)	
Bilateral total	4,074 (24.1)	3,744 (24.0)	330 (25.2)	
Tumor size, cm(mean±SD)	0.7 ± 0.6	0.7 ± 0.6	0.7 ± 0.5	0.002
Tumor size group, n(%)				0.085
≤ 10mm	14,274 (84.5)	13,134 (84.3)	1,140 (86.9)	
10~20mm	2,022 (12.0)	1,886 (12.1)	136 (10.4)	
20~40mm	588 (3.5)	552 (3.5)	36 (2.7)	
>40mm	10 (0.1)	10 (0.1)	0	
Bilaterality, n(%)	1,650 (9.8)	1,496 (9.6)	154 (11.7)	0.012
Multiplicity, n(%)	3,611 (21.4)	3,265 (21.0)	346 (26.4)	<0.001
Pathology result, n(%)				0.11
Papillary ca.	16,796 (99.4)	15,486 (99.4)	1,310 (99.8)	
Follicular ca.	84 (0.5)	82 (0.5)	2 (0.2)	
Oncocytic ca.	14 (0.1)	14 (0.1)	0	

CLN* metastasis, n(%)	1,325 (7.8)	1,205 (7.7)	120 (9.1)	0.067
RAIT†, n(%)	2,018 (11.9)	1,872 (12.0)	146 (11.1)	0.3
RAIT dose, n(%)				0.5
No	14,701 (87.9)	13,546 (87.9)	1,155 (88.8)	
Low dose	1,944 (11.6)	1,802 (11.7)	142 (10.9)	
High dose	74 (0.4)	70 (0.5)	4 (0.3)	
RAIT result				>0.9
No or minimal uptake, n(%)	2,025 (100.0)	1,877 (100.0)	148 (100.0)	
Hot uptake	0	0	0	
Recurrence, n(%)	155 (0.9)	143 (0.9)	12 (0.9)	>0.9
Recurrence site, n(%)				0.2
Local	157 (94.6)	144 (95.4)	13 (86.7)	
Distant	0	0	0	
Local + distant	1 (0.6)	1 (0.7)	0	
Survival, n(%)				0.2
Alive	10,747 (99.3)	9,929 (99.3)	818 (99.6)	
Death	78 (0.7)	75 (0.7)	3 (0.4)	
Cause of Death, n(%)				>0.9
Thyroid cancer	9 (12.2)	9 (12.5)	0	
Other cause	65 (87.9)	63 (87.5)	2 (100.0)	
Follow-up duration, months (mean±SD)	52.4 ± 40.3	53.2 ± 41.0	42.6 ± 29.3	<0.001

CLN*: Central lymph node. RAIT†: Radioactive iodine treatment.

Table S3. Clinicopathologic characteristics, treatment modalities, and outcomes of SNMTC versus FNMTC in the intermediate–high-risk group.

Characteristics	Overall N = 29,678	SNMTC N = 27,161	FNMTC N = 2,517	p-value
Sex, n(%)				<0.001
Male	6,205 (20.9)	5,574 (20.5)	631 (25.1)	
Female	23,473 (79.1)	21,587 (79.5)	1,886 (74.9)	
Age, years (mean±SD)	44.5 ± 12.6	44.4 ± 12.6	45.0 ± 12.4	0.016
Age (group), n(%)				0.13
< 55 years	23,322 (78.6)	21,374 (78.7)	1,948 (77.4)	
≥ 55 years	6,356 (21.4)	5,787 (21.3)	569 (22.6)	
Operation method, n(%)				
Open	19,387 (65.3)	17,779 (65.5)	1,608 (63.9)	
Minimal incision	3,301 (11.1)	2,997 (11.0)	304 (12.1)	
Endoscopic	458 (1.5)	430 (1.6)	28 (1.1)	
Robot	6,532 (22.0)	5,955 (21.9)	577 (22.9)	
Operation name, n(%)				0.8
Lobectomy	10,817 (36.4)	9,907 (36.5)	910 (36.2)	
Lobectomy + partial or subtotal	3,123 (10.5)	2,865 (10.5)	258 (10.3)	
Bilateral total	15,738 (53.0)	14,389 (53.0)	1,349 (53.6)	
Tumor size, cm(mean±SD)	1.2 ± 0.9	1.2 ± 1.0	1.0 ± 0.8	<0.001
Tumor size group, n(%)				<0.001
≤ 10mm	17,523 (59.0)	15,914 (58.6)	1,609 (63.9)	
10~20mm	8,807 (29.7)	8,076 (29.7)	731 (29.0)	
20~40mm	2,667 (9.0)	2,523 (9.3)	144 (5.7)	
>40mm	681 (2.3)	648 (2.4)	33 (1.3)	
Bilaterality, n(%)	6,735 (22.7)	5,990 (22.1)	745 (29.6)	<0.001
Multiplicity, n(%)	10,904 (36.7)	9,755 (35.9)	1,149 (45.6)	<0.001
Extracapsular extension	23,879 (80.5)	21,824 (80.4)	2,055 (81.6)	0.12
Pathology result, n(%)				0.027
Papillary ca.	29,092 (98.0)	26,609 (98.0)	2,483 (98.6)	
Follicular ca.	497 (1.7)	465 (1.7)	32 (1.3)	
Oncocytic ca.	89 (0.3)	87 (0.3)	2 (0.1)	
Aggressive pathology*, n(%)	420 (1.4)	390 (1.4)	30 (1.2)	0.3
CLN† metastasis, n(%)	16,846 (56.8)	15,378 (56.6)	1,468 (58.3)	0.1

LLN[‡] metastasis, n(%)	4,482 (15.1)	4,119 (15.2)	363 (14.4)	0.3
Distant metastasis, n(%)				0.6
None	29,478 (99.3)	26,976 (99.3)	2,502 (99.4)	
Synchronous	139 (0.5)	130 (0.5)	9 (0.4)	
Metachronous	61 (0.2)	55 (0.2)	6 (0.2)	
Distant metastasis organ, n(%)				0.5
Lung	168 (82.4)	156 (83.0)	12 (75.0)	
Bone	25 (12.3)	22 (11.7)	3 (18.8)	
Brain	3 (1.5)	3 (1.6)	0	
Multiple	5 (2.5)	4 (2.1)	1 (6.3)	
Other	3 (1.5)	3 (1.6)	0	
RAIT[§] , n(%)	14,215 (47.9)	13,025 (48.0)	1,190 (47.3)	0.5
RAIT dose, n(%)				0.05
No	15,204 (51.7)	13,891 (51.6)	1,313 (52.5)	
Low dose	8,571 (29.1)	7,891 (29.3)	680 (27.2)	
High dose	5,644 (19.2)	5,134 (19.1)	510 (20.4)	
RAIT result, n(%)				0.4
No or minimal uptake	14,126 (99.4)	12,946 (99.4)	1,180 (99.2)	
Hot uptake	91 (0.6)	81 (0.6)	10 (0.8)	
Recurrence	883 (3.0)	824 (3.0)	59 (2.3)	0.051
Recurrence site, n(%)				>0.9
Local	818 (90.2)	763 (90.1)	55 (91.7)	
Distant	51 (5.6)	48 (5.7)	3 (5.0)	
Local + distant	33 (3.6)	31 (3.7)	2 (3.3)	
Survival, n(%)				<0.001
Alive	19,858 (98.3)	18,151 (98.2)	1,707 (99.5)	
Death	335 (1.7)	327 (1.8)	8 (0.5)	
Cause of death, n(%)				0.5
Thyroid cancer	81 (23.2)	80 (23.5)	1 (11.1)	
Other cause	268 (76.8)	260(76.5)	8 (88.6)	
Follow-up duration, months (mean±SD)	56.0 ± 41.9	57.0 ± 42.8	45.6 ± 29.5	<0.001

Aggressive pathology[*]: Hobnail, tall cell, columnar cell, diffuse sclerosing variant. CLN[†]: Central lymph node. LLN[‡]: Lateral lymph node. RAIT[§]: Radioactive iodine treatment.

Table S4. Clinicopathological characteristics, treatment modalities, and outcomes of FNMTC based on the affected family members.

Characteristics	Overall N = 3,829	One affected N = 3,354	Two affected N = 410	Three or more affected N = 65	p-value
Sex, n(%)					0.041
Male	873 (22.8)	744 (22.2)	109 (26.6)	20 (30.8)	
Female	2,956 (77.2)	2,610 (77.8)	301 (73.4)	45 (69.2)	
Age, years (mean±SD)	45.0 ± 12.1	45.0 ± 12.2	44.9 ± 11.3	43.8 ± 11.4	0.8
Age (group), n(%)					0.083
< 55 years	2,986 (78.0)	2,597 (77.4)	337 (82.2)	52 (80.0)	
≥ 55 years	843 (22.0)	757 (22.6)	73 (17.8)	13 (20.0)	
Tumor size, cm(mean±SD)	0.9 ± 0.7	0.9 ± 0.7	0.9 ± 0.6	0.8 ± 0.6	0.087
Tumor size group, n(%)					0.8
≤ 10mm	2,749 (71.8)	2,397 (71.5)	303 (73.9)	49 (75.4)	
10~20mm	867 (22.6)	767 (22.9)	86 (21.0)	14 (21.5)	
20~40mm	180 (4.7)	158 (4.7)	20 (4.9)	2 (3.1)	
>40mm	33 (0.9)	32 (1.0)	1 (0.2)	0	
Bilaterality, n(%)	899 (23.5)	758 (22.6)	124 (30.2)	17 (26.2)	0.002
Multiplicity, n(%)	1,495 (39.0)	1,286 (38.3)	181 (44.1)	28 (43.1)	0.06
Extracapsular extension, n(%)	2,055 (53.7)	1,800 (53.7)	222 (54.1)	33 (50.8)	0.9
Pathology result, n(%)					0.13
Papillary ca.	3,793 (99.1)	3,321 (99.0)	409 (99.8)	63 (96.9)	
Follicular ca.	34 (0.9)	31 (0.9)	1 (0.2)	2 (3.1)	

Oncocytic ca.	2 (0.1)	2 (0.1)	0	0	
Aggressive pathology[*] ,n(%)	30 (0.8)	26 (0.8)	4 (1.0)	0	0.8
CLN[†] metastasis, n(%)	1,588 (41.5)	1,380 (41.1)	181 (44.1)	27 (41.5)	0.5
LLN[‡] metastasis, n(%	363 (9.5)	314 (9.4)	44 (10.7)	5 (7.7)	0.6
Distant metastasis, n(%)					>0.9
None	3,814 (99.6)	3,340 (99.6)	409 (99.8)	65 (100.0)	
Synchronous	9 (0.2)	8 (0.2)	1 (0.2)	0	
Metachronous	6 (0.2)	6 (0.2)	0	0	
Distant metastasis organ, n(%)					0.4
Lung	12 (75.0)	11 (78.6)	1 (50.0)	0	
Bone	3 (18.8)	2 (14.3)	1 (50.0)	0	
Brain	0	0	0	0	
Multiple	1 (6.3)	1 (7.1)	0	0	
RAIT[§] ,n(%)	1,336 (34.9)	1,146 (34.2)	170 (41.5)	20 (30.8)	0.011
RAIT dose, n(%)					0.037
Low dose	822 (21.6)	698 (21.0)	111 (27.1)	13 (20.0)	
High dose	514 (13.5)	448 (13.5)	59 (14.4)	7 (10.8)	
RAIT result, n(%)					0.2
No or minimal uptake	1,328 (99.3)	1,140 (99.3)	169 (99.4)	19 (95.0)	
Hot uptake	10 (0.7)	8 (0.7)	1 (0.6)	1 (5.0)	
Recurrence, n(%)	71 (1.9)	57 (1.7)	12 (2.7)	2 (3.1)	0.2
Recurrence site, n(%)					>0.9
Local	66 (93.0)	52 (91.2)	12 (100.0%)	2 (100.0)	
Distant	3 (4.2)	3 (5.3)	0	0	
Local +distant	2 (2.8)	2 (3.5)	0	0	
Survival, n(%)					>0.9
Alive	2,525 (99.6)	2,203 (99.5)	280 (99.6)	42 (100.0)	
Death	11 (0.4)	10 (0.5)	1 (0.4)	0	
Unknown	1,293	1,141	129	23	
Cause of Death, n(%)					>0.9
Thyroid cancer	1 (9.1)	1 (10.0)	0	0	
Other cause	10(90.9)	9 (90.0)	1 (100.0)	0	
Follow-up duration, months (mean±SD)	44.6 ± 29.5	44.3 ± 29.3	47.5 ± 31.1	41.7 ± 28.6	0.2

Aggressive pathology[*]: Hobnail, tall cell, columnar cell, diffuse sclerosing variant. CLN[†]: Central lymph node. LLN[‡]: Lateral lymph node. RAIT[§]: Radioactive iodine treatment.

Table S5. Clinicopathological characteristics, treatment modalities, and outcomes of FNMTC based on hereditary forms.

	**Overall N = 3,829**	**Parent /Offspring N = 1,762**	**Sibling N = 1,877**	**Parent /Offspring /Sibling N = 190**	**p-value**
Sex, n(%)					0.005
Male	873 (22.8)	407 (23.1)	405 (21.6)	61 (32.1)	
Female	2,956 (77.2)	1,355 (76.9)	1,472 (78.4)	129 (67.9)	
Age, years (mean±SD)	45.0 ± 12.1	40.9 ± 12.7	48.9 ± 10.4	44.5 ± 10.4	0.001
Age (group), n(%)					0.001
< 55 years	2,986 (78.0)	1,484 (84.2)	1,346 (71.7)	156 (82.1)	
≥ 55 years	843 (22.0)	278 (15.8)	531 (28.3)	34 (17.9)	
Tumor size, cm(mean±SD)	0.9 ± 0.7	0.9 ± 0.7	0.9 ± 0.7	0.8 ± 0.6	0.056
Tumor size group, n(%)					0.6
≤ 10mm	2,749 (71.8)	1,251 (71.0)	1,361 (72.5)	137 (72.1)	
10~20mm	867 (22.6)	406 (23.0)	414 (22.1)	47 (24.7)	
20~40mm	180 (4.7)	86 (4.9)	88 (4.7)	6 (3.2)	
>40mm	33 (0.9)	19 (1.1)	14 (0.7)	0	
Bilaterality, n(%)	899 (23.5)	358 (20.3)	494 (26.3)	47 (24.7)	0.001
Multiplicity, n(%)	1,495 (39.0)	655 (37.2)	765 (40.8)	75 (39.5)	0.085

Extracapsular extension, n(%)	2,055 (53.7)	921 (52.3)	1,041 (55.5)	93 (48.9)	0.063
Pathology result, n(%)					0.6
Papillary ca.	3,793 (99.1)	1,749 (99.3)	1,856 (98.9)	188 (98.9)	
Follicular ca.	34 (0.9)	12 (0.7)	20 (1.1)	2 (1.1)	
Oncocytic ca.	2 (0.1)	1 (0.1)	1 (0.1)	0	
Aggressive pathology* ,n(%)	30 (0.8)	15 (0.9)	12 (0.6)	3 (1.6)	0.3
CLN† metastasis, n(%)	1,588 (41.5)	783 (44.4)	728 (38.8)	77 (40.5)	0.003
LLN‡ metastasis, n(%	363 (9.5)	168 (9.5)	183 (9.7)	12 (6.3)	0.3
Distant metastasis, n(%)					>0.9
None	3,814 (99.6)	1,756 (99.7)	1,868 (99.5)	190 (100.0)	
Synchronous	9 (0.2)	4 (0.2)	5 (0.3)	0	
Metachronous	6 (0.2)	2 (0.1)	4 (0.2)	0	
Distant metastasis organ, n(%)					0.8
Lung	12 (75.0)	4 (66.7)	7 (77.8)	1 (100.0)	
Bone	3 (18.8)	1 (16.7)	2 (22.2)	0	
Multiple	1 (6.3)	1 (16.7)	0	0	
RAIT§ ,n(%)	1,336 (34.9)	541 (30.7)	729 (38.8)	66 (34.7)	0.001
RAIT dose, n(%)					0.001
Low dose	822 (21.6)	302 (17.3)	474 (25.4)	46 (24.2)	
High dose	514 (13.5)	239 (13.7)	255 (13.6)	20 (10.5)	
RAIT result, n(%)					0.1
No or minimal uptake	1,328 (99.3)	541 (99.8)	721 (98.8)	66 (100.0)	
Hot uptake	10 (0.7)	1 (0.2)	9 (1.2)	0	
Recurrence, n(%)	71 (1.9)	38 (2.2)	27 (1.4)	6 (3.2)	0.09
Recurrence site, n(%)					0.4
Local	66 (93.0)	35(94.7)	25 (83.9)	6 (100.0)	
Distant	3 (4.2)	2 (5.3)	1 (3.2)	0	
Local +distant	2 (2.8)	0	2 (6.5)	0	
Survival, n(%)					0.9
Alive	2,525 (99.6)	1,140 (99.5)	1,261 (99.6)	124 (100.0)	
Death	11 (0.4)	6 (0.5)	5 (0.4)	0	
Unknown	1,293	616	611	66	
Cause of Death, n(%)					>0.9
Thyroid cancer	1 (9.1)	0	1 (20.0)	0	
Other cause	10(90.9)	6 (100)	4 (80.0)	0	
Follow-up duration, months (mean±SD)	44.6 ± 29.5	42.4 ± 27.7	46.6 ± 30.8	44.7 ± 30.5	0.007

Aggressive pathology*: Hobnail, tall cell, columnar cell, diffuse sclerosing variant. CLN†: Central lymph node. LLN‡: Lateral lymph node. RAIT§: Radioactive iodine treatment.
